# Induction of Cytoplasmic Rods and Rings Structures by Inhibition of the CTP and GTP Synthetic Pathway in Mammalian Cells

**DOI:** 10.1371/journal.pone.0029690

**Published:** 2011-12-29

**Authors:** Wendy C. Carcamo, Minoru Satoh, Hideko Kasahara, Naohiro Terada, Takashi Hamazaki, Jason Y. F. Chan, Bing Yao, Stephanie Tamayo, Giovanni Covini, Carlos A. von Mühlen, Edward K. L. Chan

**Affiliations:** 1 Department of Oral Biology, University of Florida, Gainesville, Florida, United States of America; 2 Department of Medicine, University of Florida, Gainesville, Florida, United States of America; 3 Department of Pathology, Immunology, and Laboratory Medicine, University of Florida, Gainesville, Florida, United States of America; 4 Department of Physiology and Functional Genomics, University of Florida, Gainesville, Florida, United States of America; 5 Department of Gastroenterology, Istituto Clinico Humanitas, Rozzano, Milan, Italy; 6 Rheuma Clinic for Rheumatic Diseases, Porto Alegre, Rio Grande do Sul, Brazil; Vanderbilt University Medical Center, United States of America

## Abstract

**Background:**

Cytoplasmic filamentous rods and rings (RR) structures were identified using human autoantibodies as probes. In the present study, the formation of these conserved structures in mammalian cells and functions linked to these structures were examined.

**Methodology/Principal Findings:**

Distinct cytoplasmic rods (∼3–10 µm in length) and rings (∼2–5 µm in diameter) in HEp-2 cells were initially observed in immunofluorescence using human autoantibodies. Co-localization studies revealed that, although RR had filament-like features, they were not enriched in actin, tubulin, or vimentin, and not associated with centrosomes or other known cytoplasmic structures. Further independent studies revealed that two key enzymes in the nucleotide synthetic pathway cytidine triphosphate synthase 1 (CTPS1) and inosine monophosphate dehydrogenase 2 (IMPDH2) were highly enriched in RR. CTPS1 enzyme inhibitors 6-diazo-5-oxo-L-norleucine and Acivicin as well as the IMPDH2 inhibitor Ribavirin exhibited dose-dependent induction of RR in >95% of cells in all cancer cell lines tested as well as mouse primary cells. RR formation by lower concentration of Ribavirin was enhanced in IMPDH2-knockdown HeLa cells whereas it was inhibited in GFP-IMPDH2 overexpressed HeLa cells. Interestingly, RR were detected readily in untreated mouse embryonic stem cells (>95%); upon retinoic acid differentiation, RR disassembled in these cells but reformed when treated with Acivicin.

**Conclusions/Significance:**

RR formation represented response to disturbances in the CTP or GTP synthetic pathways in cancer cell lines and mouse primary cells and RR are the convergence physical structures in these pathways. The availability of specific markers for these conserved structures and the ability to induce formation *in vitro* will allow further investigations in structure and function of RR in many biological systems in health and diseases.

## Introduction

Over the past few decades many human autoantibodies have emerged as significant disease-specific markers for systemic rheumatic diseases [1]. These autoantibodies are mainly directed against intracellular macromolecular complexes or particles, such as nucleosomes and small nuclear/cytoplasmic ribonucleoproteins [1]. Thus, human autoantibodies have also served as useful probes for exploring subcellular structures and functions because of their unexpected specificity to novel self-antigens. Examples of significant uses of human autoantibodies in further characterization of novel subcellular structures included the identification of p80-coilin in Cajal bodies (formerly known as coiled bodies) [2] and GW182 in GW bodies [3]. Within the past few years, our laboratories identified novel human autoantibodies that recognized unique cytoplasmic structures described provisionally as rods and rings. The current study reports the identification of CTPS1 and IMPDH2 as components associated with these mammalian RR.

CTPS1 and IMPDH2 are key enzymes in the biosynthetic pathway for cytidine triphosphate (CTP) and guanosine triphosphate (GTP), respectively. CTPS1 catalyzes the rate-limiting step in generating CTP from uridine triphosphate. CTP is involved in nucleic acid and phospholipid biosynthesis and plays an important role in controlling cellular proliferation [4]. Two isoforms, CTPS1 and CTPS2, have been identified with 74% amino acid similarity. The 67 kDa CTPS1 is a target for antiviral, antineoplastic, and antiparasitic drug development. Currently available specific inhibitors of CTPS1 include Acivicin and 6-diazo-5-oxo-L-norleucine (DON) [5].

The oxidation of inosine monophosphate to xanthosine monophosphate is the rate-limiting step in the *de novo* guanine synthetic pathway catalyzed by IMPDH2, which is known to have a role in regulating cell proliferation. Due to its rate-limiting property, it has been a major target for immunosuppressive, antiviral, and cancer chemotherapy. Two isoforms of IMPDH, IMPDH1 and IMPDH2, sharing 84.0% sequence identity and 95.3% similarity have been identified and both are 56–58 kDa proteins that function as tetramers [6]. A study on the regulation of IMPDH with mycophenolic acid (MPA) showed that MPA binds to IMPDH and causes a conformational change resulting in the formation of inactive angular aggregates [7]. IMPDH2 inhibitors such as MPA, Ribavirin, and Tiazofurin are currently used for various medical conditions [8]–[11].

In the current report, inhibiting either CTPS1 or IMPDH2 using different compounds demonstrated induction of RR in a variety of cell types. More importantly, RR were identified in mouse embryonic stem cells (ESCs) and their disassembly was observed upon induced differentiation.

## Results

### Rods and rings in the cytoplasm

**Figure 1:**
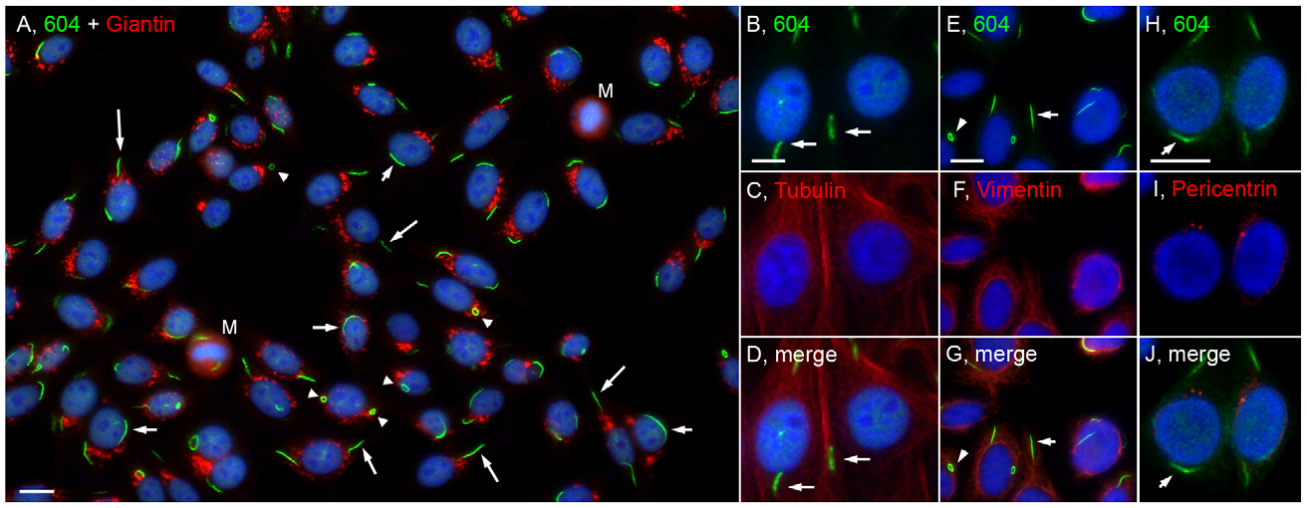
The distribution of cytoplasmic rods and rings was independent of the Golgi complex and centrosomes, and these structures were not enriched in tubulin or vimentin. (A) Merged image of HEp-2 co-stained with human anti-RR prototype serum 604/Alexa 488 goat anti-human Ig (green) and rabbit anti-giantin (Golgi marker)/Alexa 568 goat anti-rabbit Ig (red). Rods are often presented adjacent (short arrows) or perpendicular (long arrows) to the nucleus while rings (arrowheads) are found either 1 or 2 to a cell. M, mitotic cell. HEp-2 cells were also co-stained with serum 604/Alexa 488 goat anti-human Ig (green, B,E,H) and different cytoplasmic markers using mouse anti-tubulin (C), anti-vimentin (F), anti-pericentrin (I), followed by Alexa 568 goat anti-mouse Ig (red). Nuclei were counterstained with DAPI (blue). Bar, 10 µm.

**Figure 2:**
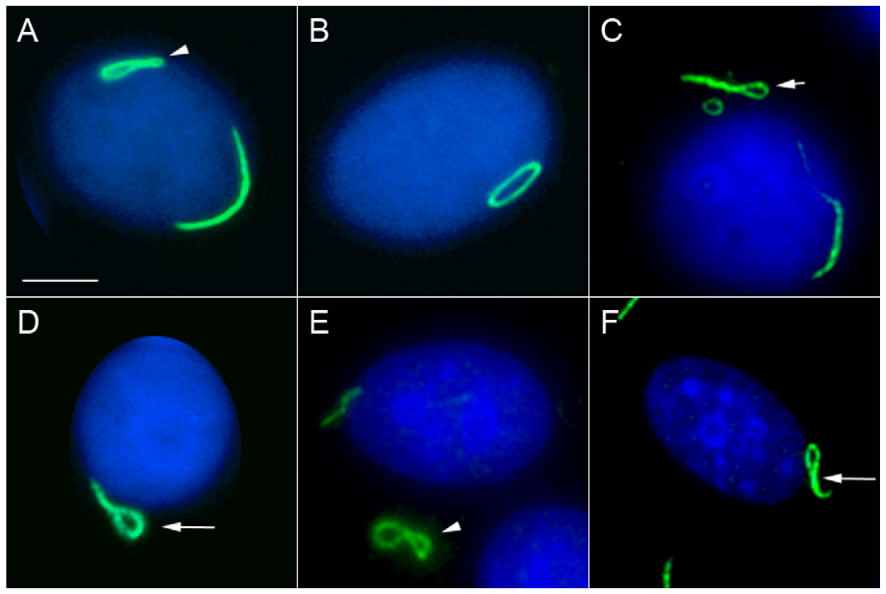
Examples of various intermediate RR structures in HEp-2 cells stained with human serum 604 (green) and counterstained with DAPI (blue). (A) Figure “8” structure (arrowhead) with a curved rod adjacent to the nuclear envelope; (B) elongated ring; (C) elongated, twisted ring (short arrow); (D) rod with a pin-loop (long arrow); (E) Figure “8” structure (arrowhead); (F) another rod with pin-loop (long arrow). Nuclei were counterstained with DAPI (blue). Bar, 5 µm.

**Figure 3:**
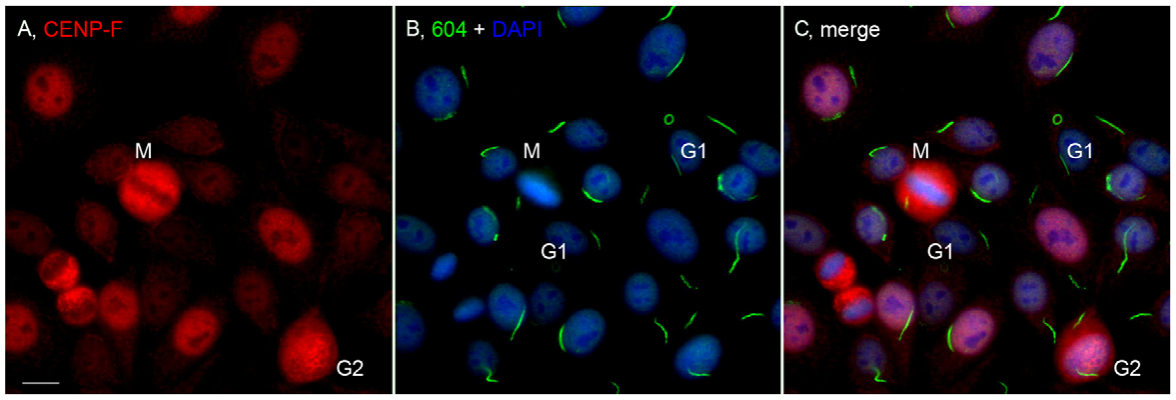
The expression of rods versus rings was not correlated with the cell cycle. HEp-2 cells were co-stained with rabbit anti-CENP-F/Alexa 568 goat anti-rabbit IgG (A) and human anti-RR serum 604/Alexa 488 goat anti-human IgG (B). G1 cells had little or no CENP-F staining, whereas late S/G2 and mitotic (M) cells showed strong staining for CENP-F. Nuclei counterstained with DAPI (blue). Bar, 10 µm.

A distinct cytoplasmic pattern was first identified in a routine antinuclear antibody (ANA) test using HEp-2 cell slides from INOVA Diagnostics Inc. These ANA slides are generally used in clinical laboratories for patients developing antibodies to self-proteins/antigens as a diagnostic screening assay for autoimmune diseases. The structures recognized by a prototype human serum 604 were distinct cytoplasmic rods (∼3–10 µm in length) and rings (∼2–5 µm in diameter, Figure 1). On average, there are one to two rods and/or rings per cell including some interesting apparent intermediate structures such as a figure “8”, an elongated ring, twisted ring, rods with pin loops at one end, as well as some that appeared to be transitioning from rods to this peculiar form (Figure 2). Immunofluorescence was performed to visualize the RR structure and location with respect to the nucleus and the Golgi complex (Figure 1A). Some rods often align adjacent to the nucleus (short arrows, ∼40% frequency) or perpendicular to the nucleus (long arrows, ∼40% frequency), and rings may be found in the cytoplasm (arrowheads, 5–10% frequency). RR are expressed in some mitotic cells; however, co-staining studies with anti-centromere protein F (CENP-F), showed that the expression of either rods or rings were independent of the cell cycle, as they appear in all stages (Figure 3). CENP-F is a cell cycle marker that has little or no expression at the G1 phase, with increasing level during the S phase, and reaching highest expression during late S and G2 phases [12], [13].

To examine the relationship of RR with other known cytoplasmic organelles, co-localization studies were performed using known markers for cytoplasmic structures. Co-staining studies with antibodies to the Golgi complex showed that RR were independent of the Golgi complex (Figure 1A). Although many rods appeared to be in the same vicinity as the Golgi complex, there are also many clear-cut examples of rods unassociated with this subcellular structure. Cytoplasmic structures, referred to as “actin rockets,” were previously described in bacteria-infected cells [14]. To determine whether RR are related to actin rockets, co-staining with mouse anti-actin antibody was performed to show that RR are not enriched in actin (data not shown). Furthermore, upon careful examination at higher magnification, the HEp-2 cells in our laboratory were not contaminated with bacteria and cell viability was >99%. Since RR are filamentous, co-staining experiments were also performed to determine whether tubulin or vimentin, was enriched in RR. Costaining showed that RR are not enriched in tubulin (Figure 1B–D). Co-staining of human prototype serum 604 and anti-vimentin antibody ruled out enrichment of RR with vimentin (Figure 1E–G).

Another initial consideration was that primary cilia, which function as nonmotile sensory organelles and have a rod-like appearance [15], could be a possible candidate. To rule out RR as primary cilia, a co-staining study with anti-pericentrin, which recognizes centrosomes, was conducted. Centrosomes are the precursors of primary cilia and are often located at the ends of the primary cilia. Since centrosomes were not located at the ends of rods recognized by human prototype serum 604 (Figure 1H–J), these rods are not primary cilia. In fact, there is no noticeable association of either rods or rings with centrosomes. Furthermore, upon closer examination, the fact that ring-type structures have never been observed for primary cilia is consistent with the conclusion.

These findings are highly reproducible using HEp-2 cell substrate from INOVA, as well as home-made cell slides prepared with inhibitor-treated cells (see below) using various commonly used fixatives in biological laboratories including acetone at −20°C for 5 minutes, acetone/methanol (1∶1) for 10 minutes, or 3% paraformaldehyde and 0.1% triton-X. Co-staining experiments with known subcellular markers showed no association with known cytoplasmic organelles such as GW bodies (data not shown).

### Identification of CTPS1 and IMPDH2 as RR components

**Figure 4:**
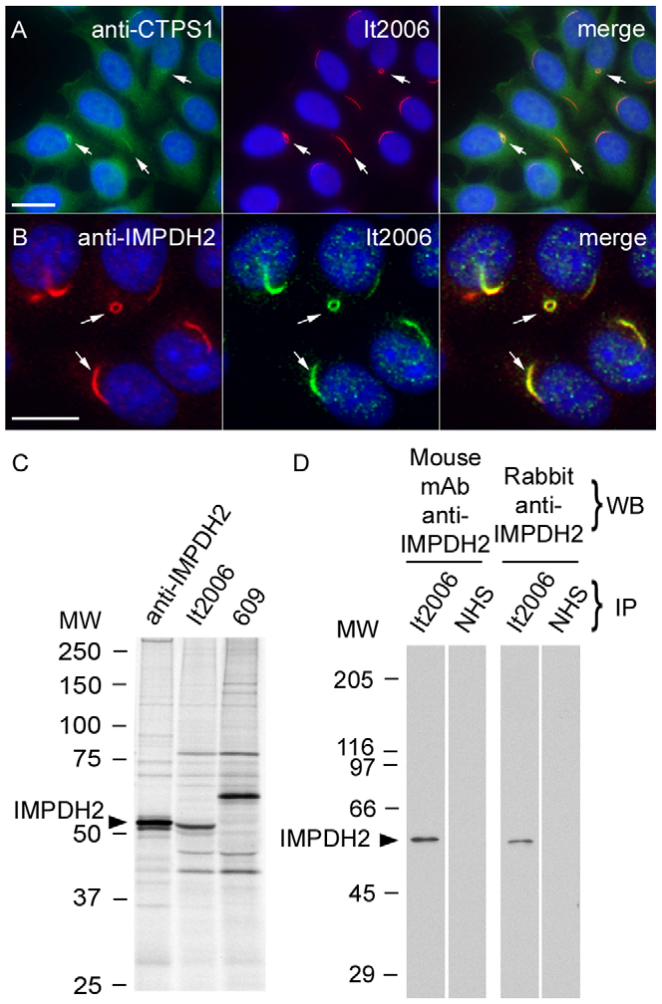
CTPS1 and IMPDH2 were highly enriched in RR and human anti-RR prototype serum It2006 recognized IMPDH2. (A) Enrichment of CTPS1, detected by rabbit anti-CTPS1 (green), to RR (arrows) identified by It2006 (red). Nuclei were counterstained with DAPI (blue). (B) IMPDH2 stained by rabbit anti-IMPDH2 (red) localized to RR (arrows) detected by It2006 (green). Bar, 10 µm. (C) Immunoprecipitation (IP) analysis using an extract of [^35^S]-methionine-labeled K562 cells and rabbit anti-IMPDH2, It2006, and a second human anti-RR serum 609. It2006 recognized a 55 kDa protein band that co-migrated with IMPDH2 immunoprecipitated by rabbit anti-IMPDH2. Serum 609 did not immunoprecipitate the 55 kDa protein. (D) IP-Western blot demonstrated that serum It2006 immunoprecipitated IMPDH2, which was recognized by both mouse monoclonal and rabbit anti-IMPDH2 antibodies. NHS, control normal human serum.

During the initial phase of our study, CTPS1 became a candidate RR protein because, in the Carnegie protein trap study, it was noted that similar rod-like structures were observed in *Drosophila* embryos carrying GFP-CTPS1 [16], [17]. Co-staining studies with rabbit anti-*Drosophila* CTPS antiserum showed localization of CTPS1 to RR, identified with prototype human anti-RR serum It2006 (Figure 4A). As there are 75.0% sequence identity and 89.8% similarity between human CTPS1 and CTPS2, antiserum to CTPS1 probably crossreacts with CTPS2 and both might be components of RR.

A second candidate RR protein was postulated in part because of an isolated ring-like structure in the cytoplasm identified by a single autoimmune serum recognizing the enzyme IMPDH2 [18] and the consideration that CTPS1 and IMPDH2 are enzymes in closely linked pathways as discussed below. In the first experiment to demonstrate whether IMPDH2 was localized to RR, a commercially available rabbit anti-IMPDH2 antibody was used to co-stain HEp-2 cells. Anti-IMPDH2 stained both rods and rings (Figure 4B, arrows). Immunoprecipitation analysis using an extract of [^35^S]-methionine-labeled K562 cells showed that rabbit anti-IMPDH2 and It2006 both pulled down a 55 kDa protein (Figure 4C). Furthermore, IP-Western analysis confirmed that the 55 kDa protein immunoprecipitated by It2006 was IMPDH2, since it was recognized by both mouse monoclonal anti-IMPDH2 and rabbit anti-IMPDH2 (Figure 4D). Since human IMPDH1 and IMPDH2 have 95.3% sequence similarity, our data cannot exclude IMPDH1 is also enriched in RR.

### Expression of rods and rings in cultured cells

**Figure 5:**
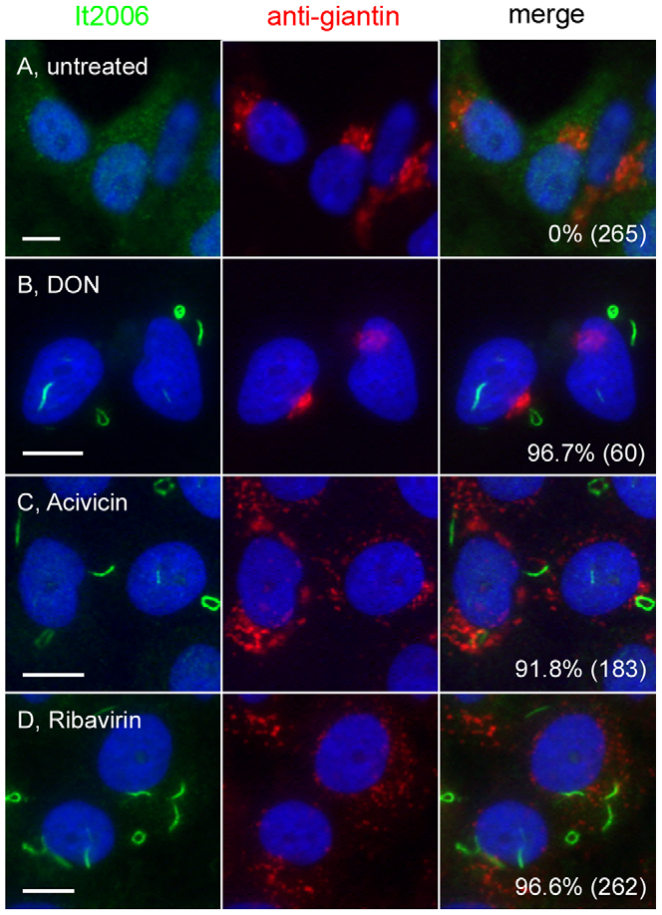
Inhibition of CTPS1- or IMPDH2-induced formation of rods and rings. Untreated HEp-2 cells (A) and cells treated with inhibitors 2 mM DON (CTPS inhibitor, B), 2 mM Acivicin (CTPS inhibitor, C), or 2 mM Ribavirin (IMPDH2 inhibitor, D) for 24 h were co-stained with human anti-RR serum It2006 (green) and rabbit anti-giantin (red). Nuclei were counterstained with DAPI (blue). The percentage of cells with RR displayed for each experiment is shown in the lower right corner with the total number of cells counted indicated in parentheses. Bar, 10 µm.

HEp-2 cell slides from INOVA Diagnostics have been consistently positive for RR, tested from lot to lot, since this study was initiated over five years ago. The same was not true for HEp-2 cell ANA slides from other manufacturers examined to date, with staining varying from completely negative to granular or diffused cytoplasmic staining. This could be the explanation for why the subcellular staining observed by Blüthner *et al* [18] was largely different from ours, except for showing one or two isolated ring structures. These differences among products from several manufacturers remains a question not completely resolved, but may stem from differences in culture conditions, proprietary sample processing, or both. Homegrown HEp-2 cells on coverslips or 8-chambered slides have generally not been positive for RR staining unless cells were kept in rapidly dividing log phase or under subconfluent cell density. Confluent cultures were generally negative for RR. Variability from experiment to experiment was clearly observed, and on average only 5–30% of cells were positive for RR detectable by serum 604 or It2006. Cells were always co-stained with both human anti-RR and rabbit anti-giantin (Golgi marker) antibodies to ensure that variables were not introduced in the fixation and immunostaining steps in these experiments. Exactly how INOVA Diagnostics can produce highly consistent HEp-2 cell slides with >95% cells positive for RR staining remains unresolved, but clearly the difference must stem from their unique manufacturing methods. Thus, HEp-2 cells do not express RR under our standard culture conditions. Cells that do not ordinarily express RR have a diffused cytoplasmic pattern when stained with prototype human serum (Figure 5A). However, it was noted that cells recently thawed from liquid nitrogen express RR in about 15% of the cells. Treating cultured cells with 0.015% to 0.5% of DMSO overnight to simulate freezing medium condition (often with 10% DMSO) did not induce formation of RR.

### Induction of rods and rings in cultured HEp-2 cells

When CTPS1 and IMPDH2 were identified as RR components, the next series of experiments focused on the effects of inhibiting these enzymes. Effects of each drug treatment were analyzed by immunofluorescence (Figure 5). The first applied was the CTPS1 inhibitor, DON, on HEp-2 cells cultured at 2 mM, a functional concentration reported in literature [5], [19]. A surprising finding was that both rods and rings were readily detected in 96.7% of cells after overnight incubation (Figure 5B) compared to 0% in untreated control cells cultured in parallel (Figure 5A). The RR detected in DON-treated cells were identical in frequency to those observed in the INOVA HEp-2 slides. A second independent CTPS1 inhibitor Acivicin [4], [5], as well as the IMPDH2 inhibitor Ribavirin, also showed the ability to induce RR formation under similar conditions (Figure 5C and D). In addition, RR induced by 2 mM DON, known to inhibit CTPS1 but not IMPDH2, were detectable with the anti-IMPDH2 antibody along with the human anti-RR serum. Therefore, CTPS1 and IMPDH2 appeared to aggregate after induction with either inhibitor to form RR.

Inhibition of CTPS1 or IMPDH2 induced RR formation in a concentration-dependent manner (Table 1). The three inhibitors had different efficiency in the induction of RR formation in HEp-2 cells. Ribavirin was the most potent inducer of RR; even at concentrations lower than 7.81 µM, Ribavirin induced RR in nearly all cells, whereas DON and Acivicin did not induce RR efficiently at these low concentrations (Table 1, p<0.0001). Significant induction of RR by Ribavirin vs DON was observed at a concentration lower than 0.5 mM (p<0.0001) and vs Acivicin at a concentration lower than 15.6 µM (p<0.0001). Induction of RR by Acivicin vs DON was significant between 0.25 mM and 15.6 µM (p<0.0001).

**Table 1:** Concentration-dependent induction of RR in HEp-2 cells using different CTPS1 and IMPDH2 inhibitors for 24 h.

	Percent HEp-2 cells with RR (total counted); number of experiments		
Drug Concentration	Ribavirin^1^	DON^2^	Acivicin^3^
2 mM^4^	98.2% (231); n = 3	94.8% (98); n = 5	97.3% (226); n = 4
1 mM^4^	98.9% (194); n = 3	87.9% (108); n = 3	98.5% (69); n = 1
0.5 mM^4^	99.2% (142); n = 2	87.2% (102); n = 2	94.3% (172); n = 3
0.25 mM^5^	98.5% (135); n = 2	28.4% (116); n = 2	94.5% (109); n = 3
125 µM^5^	94.4% (108); n = 2	29.1% (79); n = 2	98.3% (121); n = 3
62.5 µM^5^	90.8% (120); n = 2	22.9% (96); n = 2	98.1% (53); n = 2
31.3 µM^5^	98.9% (183); n = 2	23.3% (124); n = 2	95.2% (62); n = 2
15.6 µM^6^	96.8% (160); n = 2	11.6% (155); n = 2	30.4% (23); n = 2
7.8 µM^7^	94.1% (202); n = 2	5.1% (118); n = 2	0% (45); n = 2
0.2 µM^7^	95.6% (249); n = 3	2.8% (105); n = 2	0% (82); n = 1
0 µM^4^	0% (300); n = 3	0% (300); n = 3	0% (300); n = 3

1 Ribavirin induced RR in nearly all cells regardless of the tested concentration (0.2 µM–2 mM). There is no statistical significance (ns) between concentrations after Bonferroni's adjustment except with untreated control (0 µM; p>0.005);

2 DON induced RR in most cells at concentrations higher than 0.5 mM (p<0.005, 0.5 mM or higher vs any lower concentration);

3 Acivicin started to induce RR at 15.6 µM and concentrations higher than 31.3 µM, nearly all cells had RR (p<0.005 vs lower concentration) and no difference between concentrations was observed;

4 ns between Ribavirin, DON, or Acivicin;

5 Ribavirin vs DON, p<0.0001, Ribavirin vs Acivicin, ns, DON vs Acivicin, p<0.0001;

6 Ribavirin vs DON, p<0.0001, Ribavirin vs Acivicin, p<0.0001, DON vs Acivicin, p = 0.024;

7 Ribavirin vs DON, p<0.0001, Ribavirin vs Acivicin, p<0.0001, DON vs Acivicin, ns.

There are also differential time requirements for RR induction in HEp-2 cells treated with inhibitors at a fixed 2 mM concentration (Table 2). Ribavirin induced RR as early as 30 min and between 1 and 24 h, nearly all cells had RR. Induction of RR by Ribavirin was significantly faster than DON, which in turn was significantly faster than Acivicin (Table 2).

**Table 2:** Differential time requirements for RR induction in HEp-2 cells treated with different CTPS1 and IMPDH2 inhibitors at 2 mM concentration.

	Percent HEp-2 cells with RR (total counted); number of experiments		
Time after addition	Ribavirin^1^	DON^2^	Acivicin^3^
0 min	0% (300); n = 3	0% (300); n = 3	0% (300); n = 3
30 min^4^	15.2% (67); n = 2	8.3% (120); n = 1	5.2% (102); n = 1
1 h^5^	98.1% (53); n = 2	13.7% (80); n = 3	11.5% (87); n = 3
2 h^5^	95.2% (42); n = 2	20.2% (89); n = 2	19.3% (119); n = 2
3 h^6^	98.1% (55); n = 2	70.5% (88); n = 3	39.4% (109); n = 2
24 h^7^	98.2% (231); n = 3	94.8% (98); n = 5	97.3% (226); n = 4

1 Ribavirin induced RR as early as 30 min and between 1 to 24 h, nearly all cells had RR (p<0.0001, 0 min vs all time points and 30 min vs 1 to 24 h). There is no statistical significance between 1 to 24 h after Bonferroni's adjustment;

2 Induction of RR by DON was slower than Ribavirin (p<0.0001, 1, 2, and 3 h; ns, 30 min and 24 h) and the percentage of RR positive cells gradually increased after 1–3 h and was nearly all cells at 24 h.

3 Acivicin had similar pattern to DON in which RR positive cells gradually increased after 1 h and nearly all cells at 24 h.

4 Ribavirin vs DON and DON vs Acivicin, ns; Ribavirin vs Acivicin, p = 0.03;

5 Ribavirin vs DON and Ribavirin vs Acivicin, p<0.0001; DON vs Acivicin, ns;

6 Ribavirin vs DON, DON vs Acivicin, Ribavirin vs Acivicin, p<0.0001;

7 ns between Ribavirin, DON, and Acivicin.

On average, cells treated with DON or Acivicin had two RR per cell (Figure 5B, 5C), while cells treated with Ribavirin had more than two RR per cell (Figure 5D). The additive effect of two inhibitors on RR formation was also investigated. Addition of both DON and Ribavirin at 2 mM did not have a noticeable effect on RR formation since both alone induced formation of RR in >95% of treated cells. However, when inhibitors were applied at lower concentrations and for shorter periods, obvious additive effects were observed. For example, treatment of HEp-2 cells with 1 mM DON for 3 h induced RR in 52% of cells; however, when this was combined with 1 mM Ribavirin for 30 min, which alone induced RR in 13% of cells, RR was detected in 91% of cells. In addition, when HEp-2 cells were exposed to lower concentrations of Ribavirin (2 µM, 3 h), the RR structures were small and difficult to visualize; however when combined with DON (1 mM, 3 h), typical RR structures were observed. Thus, inhibition of both CTPS1 and IMDPH2 had apparent additive effects on RR formation, supporting the finding that they aggregated in the same structures. An unrelated compound, enoxacin, used as an oral broad-spectrum fluoroquinolone antibacterial agent observed to enhance miRNA activity [20] served as an additional negative control as it did not induce RR after 24 h treatment in various concentrations (10–100 nM). Thus, the inhibition of either CTPS1 or IMPDH2 appeared to be specific for the induction of RR in HEp-2 cells.

### Induction of RR in other cancer and primary cells

**Figure 6:**
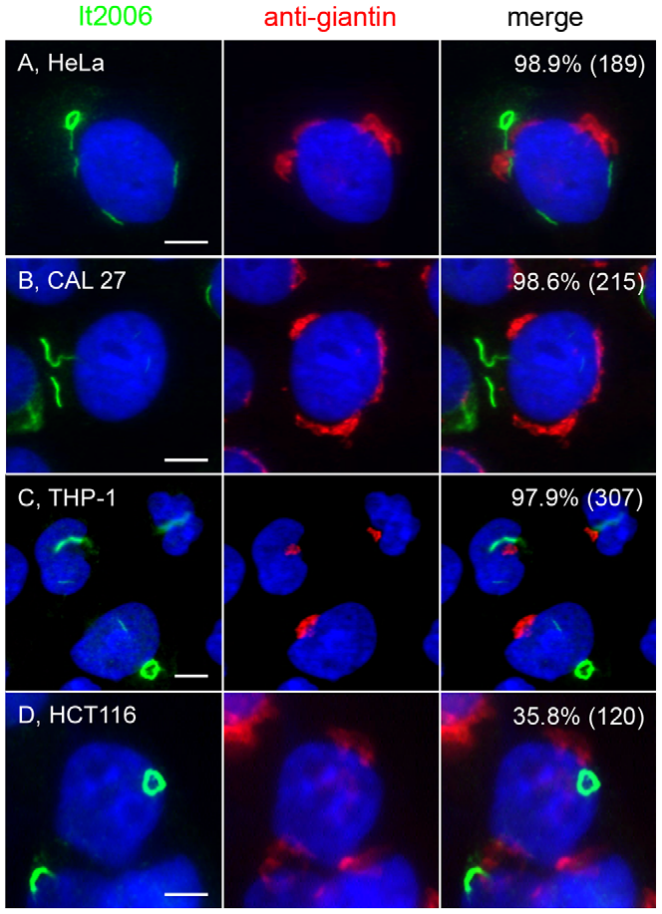
Inhibition of CTPS1-induced formation of RR in several human cancer cell lines. Induction of RR observed in human cancer cell lines HeLa (A), CAL 27 (B), THP-1 (C), and HCT116 (D) when treated with 2 mM DON in culture for 24 h, fixed, and co-stained with human anti-RR serum It2006 (green) and rabbit anti-giantin (red). Untreated controls showed few or no RR. The percentage of cells with RR displayed for each condition is shown in the upper right corner with the total number of cells counted indicated in parentheses. Bar, 5 µm.

**Figure 7:**
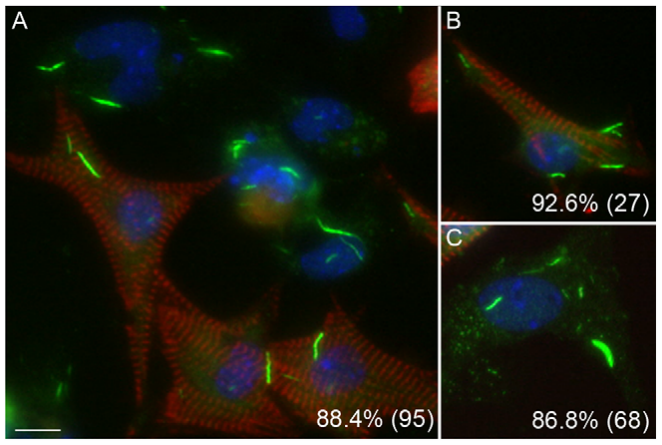
Inhibition of CTPS1-induced formation of RR in mouse primary cardiomyocytes, fibroblasts, and endothelial cells. Mouse primary cardiomyocytes prepared together with fibroblasts and endothelial cells, were treated with 2 mM DON and cultured for 24 h. Cells were co-stained with human anti-RR serum IT2006 (green) and mouse anti-actinin monoclonal antibody (red). Nuclei counterstained with DAPI (blue). Actinin-positive cardiomyocytes (A, B, red) as well as actinin-negative fibroblast or endothelial cells (A, C) all show distinct rods. The percentage of cells with RR displayed is shown in the lower right corner with the total number of cells counted indicated in parentheses (A, all cells; B, actinin-positive cardiomyocytes only; C; actinin-negative fibroblast and endothelial cells). Bar, 10 µm.

To address whether the induction of RR is conserved, multiple cell lines were analyzed, including other adherent monolayer cell lines like HeLa (Figure 6A), CAL 27 (Figure 6B), and HCT116 (Figure 6D), as well as a suspension monocytic cell line THP-1 (Figure 6C). Cells were incubated with DON for 24 h and co-stained with serum It2006 and anti-giantin. All cell lines expressed RR at a high frequency (∼98% for HeLa, CAL 27, and THP-1), comparable to the frequency observed in HEp-2 cells except HCT116 cells, with only 35.8% of cells positive for RR. Untreated cells normally did not show expression of RR, but isolated HeLa cells showed some cytoplasmic granular staining and THP-1 produced some short “immature” rods. To determine whether the expression of RR could be observed in species other than human, mouse 3T3 fibroblasts and rat NRK cells were stained with It2006. Freshly defrosted 3T3 and NRK often showed expression of RR (5 to 25% of cells) in the first one or two passages, but their expression disappeared completely in subsequent passages. The ability of freshly thawed cells to express RR was not examined further due to the lack of consistency between batches of frozen cells. Mouse primary cardiomyocytes, as well as the often co-purified endothelial cells and fibroblasts isolated from neonatal heart, did not express RR until induced with 2 mM DON in overnight culture (Figure 7). Overall 88.4% of cells had detectable RR, but cardiomyocytes detected by co-staining of actinin had up to 92.6% showing induced RR (Figure 7B). Thus, RR expression appeared to be conserved as they could be induced in multiple immortalized cells, as well as in primary cultured cells.

### Induction of RR is quantitatively sensitive to the level of cellular IMPDH2 protein

**Figure 8:**
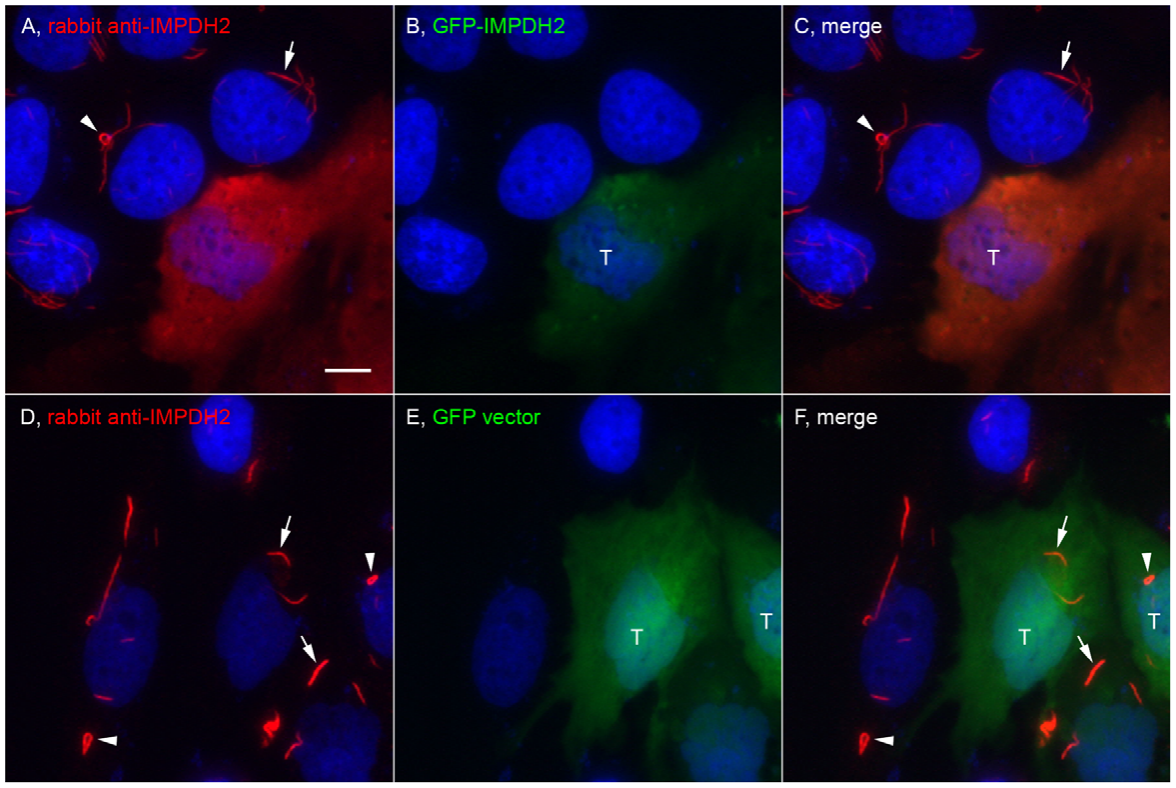
Overexpression of IMPDH2 prevented the induction of RR. HeLa cells were transfected with either GFP-IMPDH2 (A–C) or a GFP vector (D–F) for 24 h and followed by 2 mM Ribavirin incubation for 24 h. Cells were stained with rabbit anti-IMPDH2 (red) and counterstained with DAPI (Blue). GFP-IMPDH2 transfected cells (T) did not express RR while untransfected cells showed rods (arrows) and rings (arrowheads). RR were detected in both GFP vector transfected and untransfected cells. Bar, 10 µm.

To monitor the formation of RR, the use of GFP fusion constructs was explored. HeLa cells were transfected with GFP-IMPDH2 (Figure 8A–C) or the GFP vector alone (Figure 8D–F). Following transfection, cells were treated with 2 mM of Ribavirin for 24 h, and then fixed and stained with rabbit anti-IMPDH2. GFP-IMPDH2 transfected cells had brighter fluorescence compared to untransfected cells, confirming the expression of recombinant IMPDH2 (Figure 8A). Surprisingly, GFP-IMPDH2 transfected cells did not show RR after Ribavirin induction, whereas neighboring untransfected cells showed RR as expected (Figure 8C). To rule out transfection-induced artifacts interfering with the induction of RR, the induction of RR in control GFP-vector transfected cells was not affected by the transfection (Figure 8F). Series of experiments were performed to explore whether the lowest expression of transfected GFP-IMPDH2 could produce cells with GFP-labeled RR by titrating the amount of transfected plasmid and by reducing the time of transfection. None of these experiments yielded live cells labeled with GFP-RR. Therefore, the overexpression of IMPDH2 appeared to inhibit formation of RR, even at high concentrations of Ribavirin. When these GFP-IMPDH2 transfected cells were exposed to higher concentrations of Ribavirin up to 8 mM, only small aggregates were observed in some of the transfected cells.

**Figure 9:**
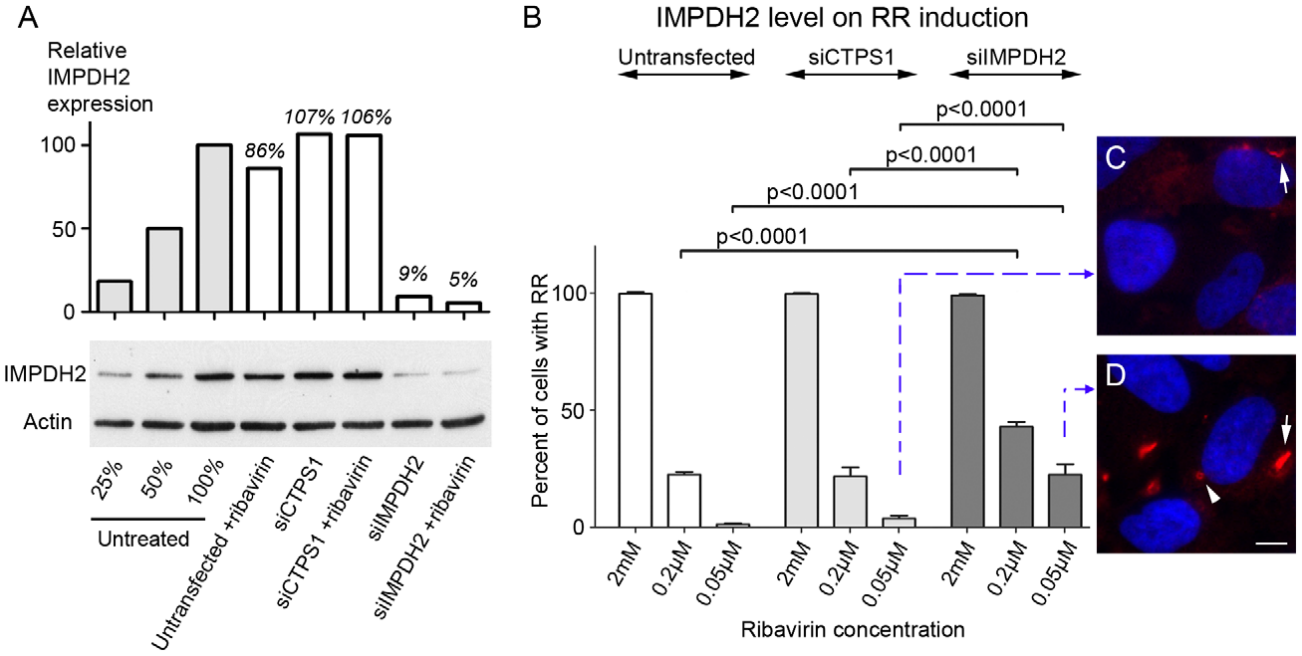
RR formation increasingly sensitive to Ribavirin when IMPDH protein level is reduced by specific siRNA knockdown. HeLa cells were either untransfected, transfected with siCTPS1, or siIMPDH2 for 44 h and followed by incubation of Ribavirin at various concentrations for an additional 4 h. (A) IMPDH2 expression was monitored by Western blot using rabbit anti-IMPDH2 antibody. Serial dilutions of untreated HeLa cell lysates (100%, 50%, and 25%) were used to help to comparatively quantitate the degree of protein knockdown. The level of actin was used as a loading control. Relative IMPDH2 protein levels were quantified using image J and normalized to the serial dilution standards. (B) Percentage of cells with RR were quantified using Mayachitra Imago with a range of 131 to 298 cells counted per data point. Cells treated with siIMPDH2 had a significant increase in percentage of cells with RR at 0.2 µM and 0.05 µM of Ribavirin analyzed by Fisher's exact test. (C–D) Representative image of cells at the lowest concentration of Ribavirin transfected with siCTPS1 or siIMPDH2. Arrows, rods; arrowhead, ring. Bar, 5 µm.

Since overexpression of IMPDH2 inhibited RR induction, the next series of experiments were to address whether knockdown of CTPS1 or IMPDH2 would affect the induction of RR. The adopted strategy was to examine cells transfected with either CTPS1 siRNA (siCTPS1) or IMPDH2 siRNA (siIMPDH2). Thus, HeLa cells were either untransfected or transfected with siCTPS1 or siIMPDH2 for 44 h, followed by an additional 4 h of incubation with three different concentrations of Ribavirin at 2 mM, 0.2 µM, or 0.05 µM. The 2 mM Ribavirin concentration was selected as the highest concentration tested and 0.2 µM was selected as the lowest based on the data presented in Table 1. Not shown in Table 1 for HEp-2 cells, 0.05 µM Ribavirin was tested in HeLa cells and was shown to be ineffective in the induction of RR. The induction of RR was monitored by double immunofluorescence using rabbit anti-IMPDH2 antibody as well as It2006. After siIMPDH2 knockdown, Western blot analysis showed reduction of IMPDH2 levels to 5–9% in untreated cells, siCTPS1 had little or no effect on the level of IMPDH2 as expected, although siCTPS1 was confirmed by real-time PCR to be functional with 62% CTPS1 mRNA knockdown (Figure 9A). The number of cells with RR were counted and plotted as a percentage (Figure 9B). While siCTPS1 transfection did not affect the percentage of cells positive for RR in all three concentrations of Ribavirin examined, knockdown of IMPDH2 showed significant increase in the percentage of cells with detectable RR formation at both 0.2 µM and 0.05 µM of Ribavirin (p = 0.0001 for 0.2 µM and 0.05 µM compared to untransfected and siCTPS1 transfected cells by Fisher's exact test). For the 2 mM Ribavirin treatment, although RR were induced in 100% of cells transfected with siIMPDH or siCTPS1, knockdown of IMPDH2 showed a decrease in the size of RR (with the average length of rod at 4.6 µm in siIMPDH2 transfected versus 6.9 µm in untransfected) along with the number of RR per cell (4.5 RR/cell in siIMPDH2 transfected versus 7.1 RR/cell in untransfected, p = 0.0024 by t-test) when compared to untransfected cells at 2 mM Ribavirin. At 0.2 µM Ribavirin, increased formation of RR was observed in cells with reduced levels of IMPDH2 (42.9% of cells in siIMPDH2 transfected vs 21.6% for siCTPS1, p = 0.0048). At 0.05 µM Ribavirin, siIMPDH2 transfected cells had 22.4% cells with shorter rods (Figure 9D) compared to siCTPS1 transfected cells with 3.6% (Figure 9C, p = 0.0001). Thus, the data show that the formation of RR by Ribavirin was sensitive to the total IMPDH2 level and excess intracellular IMPDH2 made cells resistant to the formation of RR. Therefore, RR formation appeared to depend on the molecular ratio of Ribavirin to IMPDH2 and cells with a lowered IMPDH2 level could be induced to form RR at low concentration of Ribavirin.

### RR expression in embryonic stem cells

**Figure 10:**
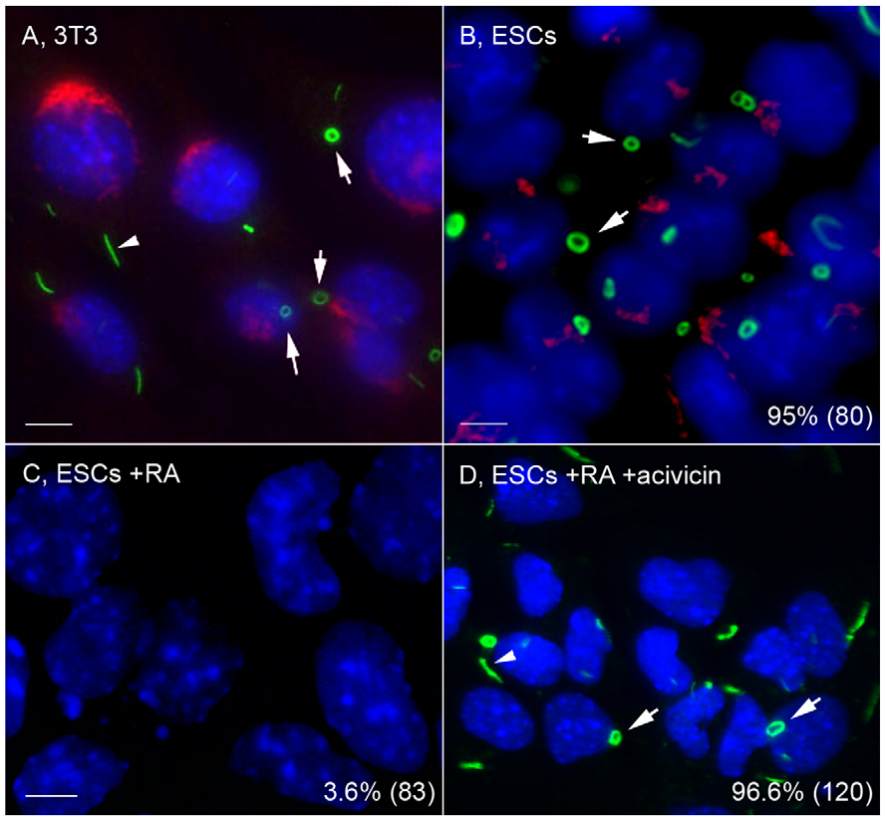
Uninduced mouse embryonic stem cells expressed predominantly cytoplasmic rings that were disassembled during retinoic acid-induced differentiation and reassembled when treated with Acivicin. (A) Mouse 3T3 cells for comparison shown with both rods (80–90%, arrowhead) and rings (10–20%, arrows). (B) Undifferentiated mouse ESCs shown with ∼90% rings (arrows) and few rods. (C) ESCs treated with RA for 4 days showed no RR. (D) RR were induced in these RA-differentiated cells by treatment with 2 mM Acivicin for 24 h. Cells were stained with human anti-RR serum It2006 (green, A–D) and co-stained with rabbit anti-giantin (red, A, B only). Nuclei counterstained with DAPI (blue). The percentage of ESCs with RR displayed for each condition is shown in the lower right corner with the total number of cells counted indicated in parentheses. Bar, 5 µm.

From the data presented in the previous sections, except for the unique case of the INOVA HEp-2 cells, various cultured cell types did not consistently express RR unless induced with one of the CTPS1/IMPDH2 inhibitors. The next question was whether certain cell types were uniquely capable of expressing RR in the absence of inducers. ESCs are highly proliferative cells that can self-renew and are pluripotent. Undifferentiated, uninduced ESCs were found to express RR (Figure 10B) similar to those described in HEp-2 cells (Figure 1A) with 1 or 2 RR per cell. Interestingly, unlike HEp-2 cells or mouse 3T3 cells (Figure 10A), where only ∼10% of cells had rings, >90% of ESC expressed rings, and <5% of cells had rods (Figure 10B). In contrast, ESCs that were differentiated with retinoic acid (RA) for 4 days no longer expressed RR (Figure 10C), but those differentiated ESCs remained capable of RR formation upon treatment of 2 mM Acivicin for 24 h and the RR phenotype was similar to the original undifferentiated ESC (Figure 10D). RR expression could be induced in multiple cancerous and primary cells, but RR were found to be expressed in untreated stem cells.

## Discussion

In this study, a novel cytoplasmic structure was identified by human autoantibodies produced by patients with chronic hepatitis C virus (HCV) infection through ANA testing. Our preliminary data showed that only a subset of HCV patients had anti-RR antibodies. Only ∼25% of HCV patients treated with both interferon-α and Ribavirin, the most common combination therapy for HCV infection, had anti-RR antibodies while none of the untreated HCV patients developed an anti-RR response. Based on our present data that primary cells exposed to Ribavirin could form RR in vitro, one can speculate that cells from patients exposed to Ribavirin will form RR. Aggregated/modified IMPDH (and CTPS) in RR in these Ribavirin treated patients may become antigenic and trigger an autoimmune response.

Most subcellular organelles disassemble prior to mitosis and reform after cell division. Although some mitotic cells were devoid of RR and thus suggesting RR might disassemble prior to mitosis, either the disassembly of RR was incomplete or the disassembly did not occur at all in many cells, since ∼50% of mitotic cells, including all stages, clearly retained RR (data not shown). At least one autoantigen, IMPDH2, recognized by the human anti-RR prototype serum It2006 was determined through IP-Western analysis. Co-localization studies determined CTPS1 to be a second component of RR. Inhibitors of the two enzymes induced formation of RR in cells that did not express RR during continuous culture. RR induction could be accomplished with low nanomolar inhibitor concentrations. Overexpression of IMPDH2 inhibited formation of RR while knockdown of IMPDH2 protein allowed for more sensitive induction of RR with ultralow concentrations of Ribavirin that normally did not induce RR. Furthermore, in HeLa cells overexpressing IMPDH2, higher concentrations of Ribavirin were not effective and could only induce remnant of RR. Therefore, the induction of RR is inhibitor-concentration dependent with lower levels of IMPDH2 favoring RR formation in the presence of low level of Ribavirin.

Since CTPS1 and IMPDH2 are major enzymes controlling the biosynthetic pathways for CTP and GTP, respectively, the formation of RR using multiple independent inhibitors of these enzymes implies that this cytoplasmic structure is linked to these cellular metabolic pathways. It is, however, acknowledged that DON and Acivicin are not completely specific for CTPS1 but are not expected to affect activity of IMPDH [21]. The observations that the formation of RR is sensitive to the concentration of IMPDH2 (Figure 9) and that RR are present in some cases in untreated cells suggest that the formation of RR is linked to the functional level of these enzymes in cellular regulation related to ribonucleotide (CTP or GTP) synthesis. Since the inhibition of CTPS1 causes formation of RR that can be detected using an IMPDH2 antibody, CTPS1 and IMPDH2 must aggregate together to form RR, although demonstration of their direct molecular interaction is lacking. Due to the large size of these structures, one may speculate that there are many other components involved in RR formation and some of these may serve as molecular linkers to facilitate interaction between CTPS and IMPDH. Future biochemical analyses are needed to explore whether there is direct interaction of CTPS and IMPDH.

### RR-like structures are evolutionarily conserved

Since our initial observation of RR over four years ago, morphologically similar structures were identified in five recent reports [17], [22]–[25] in addition to two previous reports [7], [26]. The first report of RR-like structures may be the 1987 study by Willingham *et al.* [26]. They reported a cytoplasmic “fibrillar structure” identified by an IgG3 monoclonal antibody generated from a Balb/c mouse immunized with mouse tumor cells. These structures were identified using transmission electron microscopy and immuno-electron microscopy as “large single paracrystalline arrays of individual filaments” not enclosed by a lipid bilayer membrane. Their structures were similar in appearance and number per cell to the RR in this study. The target recognized by the monoclonal antibody, an unknown 58 kDa protein in immunoprecipitation of [^35^S]-methionine radiolabeled cell lysate, was referred to as nematin (from nematodes). The identity of the antigen was not elucidated [26]. Similar to our results, they reported that these structures were unrelated to tubulin or vimentin and evolutionarily conserved in human, mouse, and rat. It was also reported that the nematin structure occurred following thawing of stored frozen cells [26] such as HEp-2, 3T3, and NRK cells, also observed in our laboratory.

The second study reporting RR-like structures was by Ji *et al.* identifying IMPDH2 protein aggregates formed from inhibition of enzymatic activity through MPA treatment [7]. The structures were identified by immunofluorescence and electron microscopy analysis [26]. Further analyses revealed that the aggregates were reversible with the addition of guanosine and its derivatives. The structures in that study and in ours were induced with two independent IMPDH2 inhibitors, MPA versus Ribavirin. Both inducers inhibit IMPDH2, but MPA is a noncompetitive and reversible inhibitor [27], while Ribavirin is a nonreversible inhibitor.

Ingerson-Mahar *et al.* demonstrated that CTPS formed filaments in *C. crescentus* [23]. Identification of the structure was through electron cryotomography along the inner curvature of *C. crescentus*. The metabolic enzyme CTPS has a bifunctional role, as a filament that regulates curvature and performs its catalytic function. A key difference was that treatment of DON in this study dissociated the CTPS filaments in bacteria, while DON induced the formation of RR in mammalian cells in our study. The latter finding on the effect of DON on mammalian cells is completely consistent with data in a recent report by Chen *et al* [25].

Other recent reports identified RR-like structures in both yeast and *Drosophila*. Noree *et al.* identified four novel filaments that were identified by screening a yeast GFP strain collection [24]. Through inhibition of CTPS, they determined that filaments were comprised of inhibited CTPS, similar to our results. In addition, they were able to visualize CTPS filaments in the *Drosophila* egg chamber, a subset of gut cells and neurons. Another report also identified an intracellular structure containing CTPS in *Drosophila* cells [17]. The structures, referred to as cytoophidia, were identified in ovary cells and other tissues [25], [28]. GFP protein trap fly stocks from the Carnegie Protein Trap Library identified CTPS as the component of cytoophidia. RR and cytoophidia may be the same conserved structures among different species [25].

Ramer *et al.* identified a large intracellular neuronal organelle that occurs in a subset of adult rat sympathetic ganglion neurons [22]. The structure, identified by immunohistochemistry plus confocal and electron microscopy, occurred mainly in a toroidal shape, but also as twists or rods. The structures, referred to as loukoumasomes or donut-like structures, were recognized by a monoclonal antibody that was raised against neuron-specific βIII-tubulin, but not recognized by other βIII-tubulin antibodies. One difference between this and our study was the increased toroidal (ring) structures in neurons, while we observed more rods than rings in all induced cancer cells, but more rings than rods in ESCs.

### Spontaneous RR versus inhibitor-induced RR

Although RR may appear to be highly conserved as discussed above, our preliminary screening in mouse liver, kidney, and spleen using indirect immunofluorescence did not detect these structures. Undifferentiated ESCs express RR without the use of inhibitors while differentiated cells do not express RR and, interestingly, differentiated cells were still capable of RR formation upon induction with a CTPS1 inhibitor. However, this should not be interpreted as de-differentiation back to stem cells. A note of caution is needed, as it is not clear whether ESCs RR are structurally and functionally the same as those of cancer cells treated with inhibitors. One clear phenotypic difference is in the ratio of rods to rings in ESCs (rod∶ring = 1∶9) compared to all other cell types with induced RR and including the RR detected in INOVA HEp-2 slides (rod∶ring = 9∶1). It is unclear whether the spontaneous formation of RR in ESCs is related to the functional activity of IMPDH2 or CTPS1 but one can speculate that, since ESCs are metabolically highly active, the spontaneous formation of RR is likely to suggest that RR-associated IMPDH2 in ESCs are functionally active. The ability to self-renew and pluripotency are two unique properties of stem cells, and these properties may have an effect on IMPDH2 (and CTPS1) activity. Once stem cells differentiate, the cellular requirement of IMPDH2 (and/or CTPS1) may change and the RR is no longer needed. One biological function of RR, as demonstrated here, is related to CTP/GTP synthetic pathways.

Similar to ESCs, the INOVA HEp-2 cells were actively dividing cells (before fixation), based on the relatively high number of detectable mitotic cells. Thus, RR in INOVA HEp-2 cells are also likely to be functional in terms of their component CTPS1 and IMPDH2. One can speculate that the requirement of CTP and GTP in rapidly dividing cells favors the aggregation of these nucleotides synthetic enzymes to possibly enhance their functional activities. This is consistent with our experience that, while freshly thawed cells can demonstrate up to 25% cells with RR in first or second cell passages, cell confluence is often associated with the complete loss of RR expression – IMPDH2/CTPS1 no longer assemble in the cytoplasm. This argues for the formation of RR to be associated with a high requirement of intracellular CTP/GTP and the associated biosynthetic enzymes are assembled into RR, which can enhance their synthetic activity.

In contrast, inducible RR in cancer cells, as well as primary cells, are composed of inactivated enzymes based on the presence of an inhibitor added to induce these structures. Higher expression of IMPDH2 inhibited formation of RR by comparable levels of inhibitors. Lower expression of IMPDH2, as in the partial knockdown experiment, promoted formation of RR with a lower concentration of inhibitor. The apparent difference between ESCs with likely active RR-associated IMPDH2/CTPS1 versus the inhibitor-induced RR with inactive enzymes may be explained as follows. When HEp-2 cells are exposed to an inhibitor, such as Ribavirin, the lower production of GTP may trigger the response for the biosynthetic enzyme to assemble into oligomer/polymer configuration to favor an increase in enzyme activity, consistent with the speculation above that ESCs have a strong GTP/CTP requirement to maintain active cell proliferation. Once RR is formed, Ribavirin – an irreversible inhibitor, “locks” the configuration of RR, making the enzymes inactive.

In summary, RR formation is regulated by the inhibition of CTPS1 and IMPDH2, important in purine and pyrimidine biosynthesis and critical for RNA and DNA synthesis. The importance of these enzymes in RNA and DNA synthesis suggests that RR may play a role in cell division. In addition, both CTPS1 and IMPDH2 aggregate together to form the RR structure and inhibition of one will cause the aggregation of the other component with RR. Studies have demonstrated that CTPS1 and IMPDH2 are aberrantly expressed in different cancers [4], [29]. However, as CTPS1 and IMPDH2 are differentially expressed, the formation of RR in cells may help regulate their overall activities. Further characterization and understanding of RR may lead to a new biomarker for diseases or novel markers for stem cells.

## Materials and Methods

### Cell culture

HeLa (human cervical cancer), HEp-2 (human epidermoid larynx carcinoma), and HCT116 (human colon cancer) were cultured in DMEM containing 10% Fetal Bovine Serum (FBS). Human oral cancer cells CAL 27 were cultured in DMEM containing 10% FBS and 1.5 g/L sodium bicarbonate. THP-1, human monocytes, and K562, human erythroleukemia cells, were cultured in RPMI 1640 with 25 mM HEPES, and 10% FBS. All cells were cultured in a 37°C incubator with 5% CO_2_. Adherent cell lines were maintained at 50% confluence. Suspension cells were maintained at an optimum concentration of 10^6^ cells/mL. All media contained 100 I.U./ml penicillin and 100 µg/ml streptomycin.

### Embryonic stem cell culture and differentiation

The murine R1 ESCs were obtained from Dr. Andre Nagy (University of Toronto) and maintained according to Hamazaki *et al.* [30]. ESCs were differentiated with 1 µM retinoic acid for 4 consecutive days as described [31].

### Mouse cardiomyocyte collection and preparation

The mouse cardiomyocytes were harvested and prepared according to Chan *et al.* [32].

### Human autoantibodies

Autoantibodies with reactivity to RR were selected by authors GC and CAvM during routine ANA screening assay. All three human anti-RR sera used in this study were from patients with chronic HCV infection and were de-identified samples. These sera were selected largely due to their relative specificity in RR staining and in available quantity to allow further analyses. Serum It2006 provided by GC, and serum 604 and 609 provided by CAvM, are considered prototype sera for anti-RR. The clinical characterization of these patients and others with anti-RR will be summarized in a subsequent report [33].

### Immunoprecipitation

Immunoprecipitation of [^35^S]-methionine-labeled K562 cells for the analysis of the proteins recognized by human autoimmune sera was performed as described [34].

### Indirect immunofluorescence

HEp-2 cell slides (INOVA Diagnostics, San Diego, CA) or various adherent cell types cultured in 8-well chamber slides (BD Falcon) were used for indirect immunofluorescence as described [35], [36]. The 8-well chamber slides were prepared using ∼60,000 cells per well in 500 µL of media. Non-adherent cells were prepared using the cytospin method to transfer cells onto glass slides as described [37]. Fixation methods included acetone at −20°C for 5 min, acetone/methanol (3∶1) at −20°C for 10 min, and 3% paraformaldehyde in PBS at room temperature for 10 min followed by 0.1% triton-X/PBS for another 10 min. For co-staining studies the following primary antibodies were used: rabbit anti-giantin [38], anti-pericentrin (a gift from Dr. Marvin Fritzler, University of Calgary), anti-Drosophila CTPS1 and pre-immune serum (a gift from Dr. Jim Wilhelm, University of California, San Diego), anti-IMPDH2 (Abcam, Cambridge, MA: ab75790; Proteintech, Chicago, IL: 12948-1-AP), mouse anti-actin, anti-actinin (Sigma-Aldrich, St. Louis, MO: A7811), anti-tubulin, and anti-vimentin, and human anti-RR serum It2006, 604, and 609. To validate that the rabbit anti-Drosophila CTPS1 serum recognized human CTPS1, a full-length human CTPS1 cDNA (IMAGE id 3355881, OpenBiosystems, Thermo Scientific) was used as a template in an *in vitro* transcription and translation reaction (TNT, Promega) to generate [^35^S]-methionine labeled translation products. The latter was shown to be immunoprecipitated by the rabbit anti-Drosophila CTPS1 serum and not by the pre-immune serum from the same rabbit.

### Induction of RR formation using CTPS or IMPDH inhibitors

Acivicin (Enzo Life Sciences, Ann Arbor, MI; BML-El113-0010) and Ribavirin (Sigma-Aldrich; R9644) were solubilized in water to 100 mM and 50 mM stock concentration, respectively. 6-diazo-5-oxo-L-norleucine (DON, Sigma-Aldrich; D2141) was solubilized in DMEM to a stock concentration of 100 mM. Cells were seeded as monolayers and allowed to attach for 24 h. Subsequently, CTPS1 or IMPDH2 inhibitors were added to various final concentrations from 0.2 µM to 2 mM and were kept in a cell incubator for times ranging from 15 min to 48 h.

### siRNA knockdown

All siRNAs used in this study were obtained from Dharmacon RNA Technologies (Lafayette, CO). The siRNAs were dissolved in molecular biology grade water and the resulting 20 µM stock was stored in aliquots at −80°C before use. The two predesigned siRNAs were: ON-TARGET plus SMART pool siRNA Human CTPS1 (target sequences: UAACAUUGAUGCAGGAACA, AGACUAAACCUACCCAGAA, GGUGUUAUAUCAGGAAUUG, and GUAUGCAGGUGCUCAAAUC), siGENOME SMARTpool siRNA IMPDH2 (target sequences: GGACAGACCUGAAGAAGAA, GCACGGCGCUUUGGUGUUC, GGAAAGUUGCCCAUUGUAA, and CUAAAGAAAUAUCGCGGUA). Transfection was performed using Lipofectamine 2000 (Invitrogen, Carlsbad, CA) per the manufacturer's instruction. The siRNA was used at a final concentration of 100 nM. Knockdown efficiency was determined by real-time PCR analysis using CTPS primers (Applied Biosystems, Hs01041851_m1) or IMPDH2 primers (Applied Biosystems, Hs00168418_m1) plus Western blot for protein expression.

### Transfection of GFP plasmid

HeLa cells were seeded in 8 well chambers at 50–70% confluency. Cells were transfected with 0.35 µg GFP-IMPDH2 (RG202977, OriGene, Rockville, MD) or a GFP vector alone using 0.53 µg of Lipofectamine 2000. The transfected cells were examined after 24 h directly as live cells or were processed for indirect immunofluorescence.

### Western blotting

Protein levels were analyzed as described [39] using rabbit anti- CTPS (Abcam, 1∶1000) or rabbit anti-IMPDH (Santa Cruz Biotechnology, Paso Robles, CA, 1∶1000). For IP-Western, IgG was crosslinked to protein A Sepharose beads as described [40] and human It2006 was used for immunoprecipitation and mouse monoclonal and rabbit anti-IMPDH from Santa Cruz Biotechnology were used for detection. Mouse anti-actin antibody 31G9 was used at 1∶5,000 or ∼1 µg/ml as a normalizer to control for loading.

### Statistical analysis

Cell counting was performed using Mayachitra Imago (Mayachitra, Santa Barbara, CA) to detect nuclei counterstained with DAPI. Unpaired, two-tailed Student's t-test and Fisher's exact test were used to compare independent groups. When appropriate, Bonferroni's adjustment for multiple comparisons was applied. For all statistical analysis, Prism for Windows, version 5.0 (GraphPad Software, San Diego, CA) was used.
